# Current Advances in DNA Methylation Analysis Methods

**DOI:** 10.1155/2021/8827516

**Published:** 2021-03-20

**Authors:** Ehsan Khodadadi, Leila Fahmideh, Ehsaneh Khodadadi, Sounkalo Dao, Mehdi Yousefi, Sepehr Taghizadeh, Mohammad Asgharzadeh, Bahman Yousefi, Hossein Samadi Kafil

**Affiliations:** ^1^Department of Agronomy and Plant Breeding, Tabriz Branch, Islamic Azad University, Tabriz, Iran; ^2^Department of Plant Breeding and Biotechnology, University of Zabol, Zabol, Iran; ^3^Drug Applied Research Center, Tabriz University of Medical Sciences, Tabriz, Iran; ^4^Faculté de Médecine, de Pharmacie et d'Odonto-Stomatologie (FMPOS), University of Bamako, Bamako, Mali; ^5^Stem Cell Research Center, Tabriz University of Medical Sciences, Tabriz, Iran; ^6^Infectious and Tropical Diseases Research Center, Tabriz University of Medical Sciences, Tabriz, Iran; ^7^Biotechnology Research Center, Tabriz University of Medical Sciences, Tabriz, Iran

## Abstract

DNA methylation is one of the epigenetic changes, which plays a major role in regulating gene expression and, thus, many biological processes and diseases. There are several methods for determining the methylation of DNA samples. However, selecting the most appropriate method for answering biological questions appears to be a challenging task. The primary methods in DNA methylation focused on identifying the state of methylation of the examined genes and determining the total amount of 5-methyl cytosine. The study of DNA methylation at a large scale of genomic levels became possible following the use of microarray hybridization technology. The new generation of sequencing platforms now allows the preparation of genomic maps of DNA methylation at the single-open level. This review includes the majority of methods available to date, introducing the most widely used methods, the bisulfite treatment, biological identification, and chemical cutting along with their advantages and disadvantages. The techniques are then scrutinized according to their robustness, high throughput capabilities, and cost.

## 1. Introduction

Epigenetics is a broad concept used to describe various reversible genomic changes [1]. Epigenetics, in the literal sense of “Beyond Genetics,” refers to inherited changes in gene expression, which result from changes in chromosomes without altering the DNA sequence. Epigenetic mechanisms of gene regulation include DNA methylation, covalent changes in histones, and noncoding and messenger RNAs [2, 3]. DNA methylation, the covalent changes in cytosine, is one of the most widely studied changes in the field of epigenetics and provided a molecular mechanism through which the expression of the gene can be regulated [4, 5]. This process is capable of preserving and transmitting epigenetic information through the replication of DNA and cell division [6]. DNA methylation is associated with a wide range of biological processes, including deactivation of chromosome X, genomic imprinting, stem cell differentiation, gene expression control, and chromosomal stability [7]. These findings indicate that DNA methylation is one of the most important modifications which play an essential role in regulating the growth of the cells and their proliferation.

Under the catalysis of the MTase methyltransferase enzyme, the fifth carbon in the 5′-CG-3′ dinucleotide can selectively be replaced by a methyl group to form 5-methyl cytosine. About 60-90% of 5′-CG-3 dinucleotides are methylated in the eukaryotes, and the nonmethylated 5′-CG-3 dinucleotides often accumulate in the promoter of structural genes and the starting point for their transcription to form CpG islands [8]. The methylation of non-CpG regions in CHH and CHG (H = A, C, T) is seen in the embryonic stem cells and in the plants [9]. The DNA methylation pattern is hereditary and mutable [10]. The normal expression of the parental allele results in the correct expression of the gene, while abnormal DNA methylation in the parental allele typically causes different cancers, genetic diseases, and aging disorders [8, 11]. Therefore, performing research on DNA methylation plays a critical role in medicine, biological sciences, and biochemistry.

Given its importance in early development and aging, DNA methylation is a crucial epigenetic modification to profile. The detection of DNA methylation patterns is a rapidly advancing area of research, which promises the possibility of the methylation profiling to distinguish various tumor and cancer types, and possibly their response to chemotherapeutic agents. Detection of aberrancies of DNA methylation appears to be one of the most significant tests in early cancer diagnosis. These important findings regarding DNA methylation would not have been possible without the advancement of various profiling approaches. The accelerated development of array and sequencing technologies has significantly improved DNA methylation profiling, providing an unprecedentedly comprehensive view of the DNA methylation landscape. The present study will serve as an introduction to methods for DNA methylation analysis. This review provides an overview of the major profiling approaches, with a focus on the recent and promising genome-wide methodologies.

## 2. Techniques Based on Bisulfite Treatment

**Figure 1:**
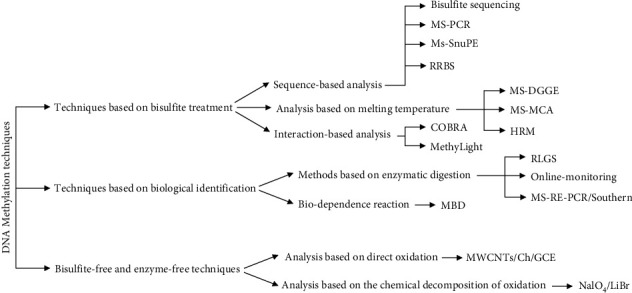
Schematic diagram of DNA methylation methods.

**Figure 2:**
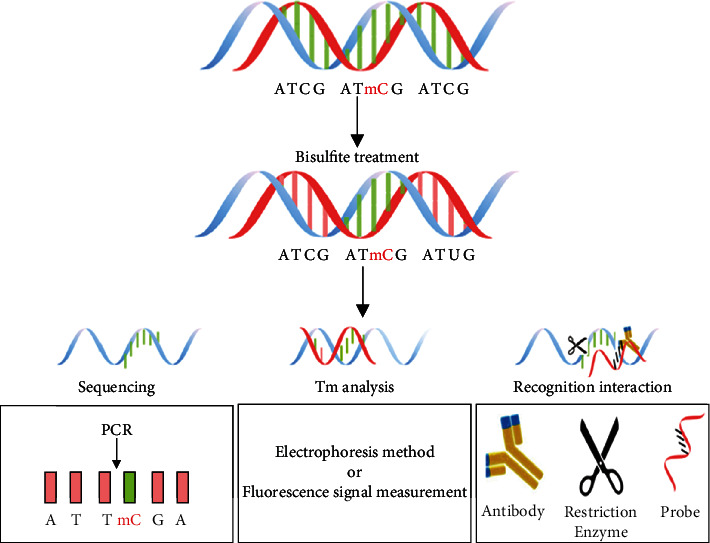
The main method for 5-methyl cytosine analysis after treatment with bisulfite.

Initial studies on DNA methylation focused on identifying the position of methylation of the examined genes and determining the total content of 5-methyl cytosine [12]. Bisulfite treatment is one of the widely used and effective methods for categorizing 5-methyl cytosine and nonmethylated bases [8]. Exposing the genomic DNA to sodium bisulfite induces nonmethylated cytosine (C) deamination and converts it to uracil (U), while the methylated cytosine remains intact. Uracil is finally converted to thymine (T) following the polymerase chain reaction (PCR) [13]. As a result, the gene methylation information is transmitted to the sequence information. The gene features, such as melting point (temperature) and specific identification interactions, change due to the sequence changes (Figure 1). The differences between these features form the basis of the DNA methylation analysis (Figure 2).

### 2.1. Sequence-Based Analysis

The gene sequencing is a simple idea to differentiate 5-methyl cytosine from other bases. In 1992, Frommer proposed the bisulfite sequencing method to determine 5-methyl cytosine in the single strands. First, the DNA is treated with sodium bisulfite and multiplied by PCR. By determining the sequence of the resulting product, the 5-methyl cytosine position is detected. This technique is suitable for determining the multiple DNA methylation, while the need for cloning and sequencing processes makes the preparation process boring and long-lasting [14]. In 1996, Herman et al. developed an advanced sequencing-based technique called methylation-specific PCR (MS-PCR) [15]. In this method different from the previous bisulfite sequencing method, two specific promoters are predesigned according to the methylated and nonmethylated sequences. Following the treatment with sodium bisulfite, the target sequence is proliferated by PCR with two specific promoters. If the target sequence is methylated, there will be multiplied 5-methyl cytosine in the PCR products. This technique avoids the complex sequencing process. However, due to the need for the sequence primer design and the necessity for recognizing the position of methylation, this technique can be only used for qualitative determination [16]. A technique called methylation-sensitive single-nucleotide primer extension (Ms-SnuPE) was developed by Gonzalgo and Liang in 1997 to overcome the limitations of MS-PCR [17]. In this method, after treatment with bisulfite, the DNA is amplified by PCR and isolated by electrophoresis. The resulting products are then used as templates for the development of the primer [18, 19]. By electrophoretic separation and analyzing the expanded end product radiation, the C/T ratio is easily obtained and the relative ratio of methylated and nonmethylated sequences is achieved [20]. This method is not only suitable for the identification of the methylation position of each gene site but also able to determine the level of methylation. However, the tedious nature of primer design and the radioactive contamination will inevitably remain as constraints [9, 21]. By studying further in sequencing methods, the techniques based on new generation sequencing were developed and used as powerful analysis methods. In 2005, Meissner et al. proposed the reduced representation bisulfite-sequencing method (RRBS) [22], in which, the Msp I restriction enzyme is used for digesting the genome to enrich the CpG sites. Then, the sequencing process is done to obtain the methylation information of every single base. RRBS, which integrates the Msp1 restriction enzyme digestion, the bisulfite conversion, and the new generation of sequencing to analyze the methylation patterns of certain components, is more cost-effective than the WGBS since the focus of these methods is on enriching the regions rich in CpG near the identification sequence of the restriction enzyme [23–25].

The bisulfite sequencing of the whole WGBS genome (MethylC-seq; BS-seq) theoretically covers all the cytosine information [26]. In this method, the genomic DNA is purified and divided into fragments. The ends of the fragmented DNAs are restored; the adenine bases are added to the end of the 3′ (tail-A) of the DNA fragments, and the methylated adapters are attached to the DNA fragments [27]. The DNA fragments are selected in terms of size before sodium sulfite treatment and PCR amplification, and the resulting library is sequenced [28]. It should be noted that a large number of PCR cycles and inappropriate selection of DNA polymerase insensitive to uracil may lead to overrepresentation of methylated DNA data [29]. Starting with an adequate amount of genomic DNA can prevent the loss of information from the studied areas and excessive reproduction [13]. The main advantage of WGBS is its ability to assess the state of methylation almost from every CpG site, including the low-density CpG regions such as the “genetic deserts” between the genes, the incomplete methylated domains, and the final regulatory elements [29]. It can also determine the absolute level of DNA methylation and reveal the sequence methylation context.

### 2.2. Analysis Based on Melting Temperature

Bisulfite sequencing provides reliable support for extensive mapping of the genome; however, multiple operations and lower credible results due to complicated bases and incomplete bisulfite conversion are challenging [30]. Therefore, the idea of identification without sequencing and analysis for the unique melting temperature of the 5-methyl cytosine sequence drew the attention. In 1999, Aggerhoim developed a methylation-specific denaturing gradient gel electrophoresis (MS-DGGE) to determine the level of 5-methyl cytosine [31]. The DNA sequences treated with bisulfite are treated with DGGE, in which the solubility of the denaturing substance increases with the melting temperature from top to down [31]. The DNA strands are converted to dendrite and stopped in different areas of the gel due to the unique temperature of methylated and nonmethylated DNA strands. Depending on the isolation result, the 5-methyl cytosine level is measured [9]. This technique is cost-effective and can provide a comprehensive analysis of DNA methylation [32, 33]. However, the separation efficiency should be improved. In 2001, the methylation-specific melting-curve analysis (MS-MCA) was designed to determine the position of 5-methyl cytosine [34].

After treatment with bisulfite, the DNA strands are labeled with a fluorescence color. Depending on the measurement of the fluorescence intensity during melting, the melting curve of the DNA strands is obtained and their melting point is determined by optical cycle processing [32]. Since the melting point has a direct relationship with the CG value in the DNA sequence, the melting temperature of the nonmethylated DNA strand is lower than the methylated DNA strand due to the conversion of CG to UG during the bisulfite treatment [35]. Therefore, the DNA methylated and nonmethylated strands are separated from each other. However, it is difficult to accurately determine the state of methylation of each of the bases by using this technique. Rodríguez López et al. proposed the high-resolution melting (HRM) method to overcome this limitation in 2010 for the direct detection of 5-methyl cytosine [36]. This approach is different from MS-MCA. In HRM, saturated fluorescent color is used to label the DNA sample. A clear change in the fluorescence signal was achieved during the melting due to the capture of the target base position by the colors [37]. With high resolution, the methylation status of each of the bases was precisely determined [38].

### 2.3. Interaction-Based Analysis

Despite the analysis of melting temperatures, some certain interactions can be considered as alternatives for economic and efficient identifying the target sequence. Restriction enzymes and oligonucleotide probes are widely used in these interactions, which are the core of this approach [39, 40]. The combined bisulfite-restriction analysis (COBRA) was introduced to determine the status of 5-methyl cytosine [8]. After treatment with bisulfite and PCR, the BstU I restriction enzyme is added to the DNA sample, which detects and cuts the methylated 5′-CGCG-3′ sequence [8]. The reaction products are then analyzed by electrophoresis to determine the status of 5-methyl cytosine [41]. This technique can easily provide accurate results. However, its scope of use is limited due to the dependence on the restriction enzyme sequence [42]. At the beginning of the 21^st^ century, enzymatic digestion technologies and oligo-probe protein hybridization were combined for the analysis of 5-methyl cytosine [43]. The MethyLight is the typical method. A specific oligo probe, Tagus, is designed and labeled with fluorophore and extinguisher for the 5′ and 3′ ends, respectively [44]. After bisulfite treatment, the DNA sample is incubated with the probe and analyzed using the real-time PCR [45]. The duplex strands are cut in 5-methyl cytosine by the restriction enzymes, and the fluorophore of the 5′ end is released. The 5-methyl cytosine level is measured from the produced fluorescence signal [46]. However, the use of restriction enzyme and the real-time PCR has made this method uneconomic and difficult.

## 3. Techniques Based on Biological Identification

**Figure 3:**
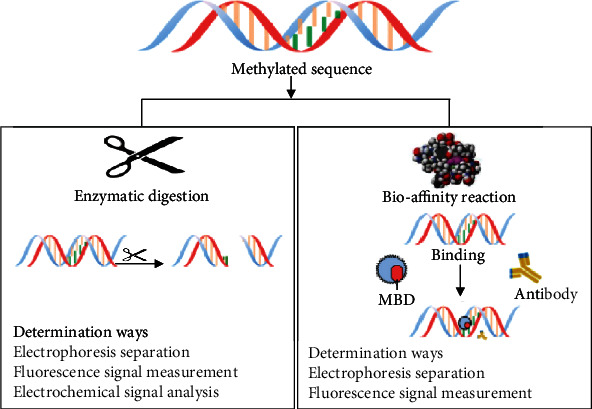
The main biological detection approach for 5-methyl cytosine and the method to determine identified targets.

Although the treatment with bisulfite is widely used to detect 5-methyl cytosine from other cytosines, it involves some issues such as incomplete conversion, false-positive results, and difficult and time-consuming use [44, 47]. In comparison, the biological identification methods, such as enzymatic digestion or biological reactions, can quickly detect the methylation with high specificity under mild reaction conditions [48]. Therefore, the 5-methyl-cytosine analysis methods based on biological identification have turned into the new center of attention (Figure 3).

### 3.1. Methods Based on Enzymatic Digestion

The methods based on enzymatic cutting use of different cutting (cleavage) characteristics of isoschizomers and nonisoschizomers. A pair of isoschizomers identify an identical sequence and have the same cutting point but show different sensitivity to the state of DNA methylation [13]. Methylation-sensitive restriction enzymes (MREs), including BstU I, Hpa II, Not I, and SmaI, only cut the nonmethylated target regions, and the methylated DNA stays intact [49]. The MRE cuttings are associated with the sequencing technologies to predict the DNA methylation levels at the genome level. In the MRE cutting sequence along with sequencing (MRE-seq), the MRE cuts the nonmethylated CpG regions in the genomic DNA and the resulting DNA fragments are selected and sequenced based on size [50]. The sequencing results reveal the location of nonmethylated CpG regions within the enzyme identification sites [51]. The MRE-seq provides the possibility of relative estimation of DNA methylation levels but has a relatively small coverage at the genome level since the identification sites containing CpG are limited [52, 53].

Since the beginning of the 21^st^ century, enzyme-related techniques have been widely used in DNA methylation studies. The routine method is the restriction-landmark genomic scanning (RLGS) approach [54]. DNA samples are digested by the Not I restriction enzyme, leading to the identification of methylated sites. The sequences are then labeled with 32P-dCTP and 32P-dGTP and digested with the methylation-insensitivity restriction enzyme (EcoR V) [55]. After initial separation, the product is again treated with another methylation-insensitivity restriction enzyme (*Hinf* I) with a high frequency by performing one-dimensional electrophoresis and subjected to two-dimensional electrophoresis [56]. After two stages of screening, the methylation status of several CpG sites is examined. However, the use of the technique is complex and the result is uncertain [57]. An advanced enzymatic digestion technique, called methylation-sensitive restriction endonuclease-PCR/southern (MS-RE-PCR), was developed [58].

The DNA samples are treated with *Hpa* II and *Msp* I restriction enzymes. Both the *Hpa* II and *Msp* I enzymes can specifically detect the 5′-CCGG-3 sequences [59, 60]. The *Hpa* II and *Msp* I are unable to cut the nonmethylated C in the 5′-CCGG-3, while the *Msp* I can cut the methylated C in the 5′-CmCGG-3′. The digested DNA samples are analyzed with *Hpa* II and *Msp* I enzymes using the PCR or southern blot, and the status of DNA methylation is obtained in several methylation sites. This is a simple and cost-effective method, but not suited for complex gene samples [61]. In 2007, the methylation-sensitive restriction endonuclease was used to monitor the online activity of MTase and examine the methylation status [62]. In this method, a molecular beam radius labeled with a pair of fluorophore and extinguisher is used as the oligo probe. The probe first shows a weak fluorescence signal in its pinhead. Under the Dam MTase catalysis, the 5-methyl cytosine fragments are produced in the probe. The addition of *Dpn* I endonuclease then cuts the strand specifically at the methylated site and increases the fluorophore signal due to the separation of the fluorophore and the extinguisher [63]. Therefore, the MTase activity and the DNA methylation status are controlled by a clear fluorescence signal. Nevertheless, the procedure and the cost of fluorescent labeling are the weaknesses of this technique.

### 3.2. Biodependence Reaction

Some biomolecules such as MBD, anti-5-methyl-cytosine IgG1 antibodies, and Zinc Finger Proteins (ZF) show the inherent ability of the methylation site-specificity and can be directly used to decompose the DNA methylation [64]. The results of the MBD-based approach, which is based on the ability of MBD proteins to specifically bind to the methylated DNA sequences, can be represented using the microarray (MBD-chip) or sequencing technologies (MBDCap-seq/MethylCap-seq) [13]. Concurrently, an MBD-based approach to screen and identify the 5-methyl cytosine in the genome was introduced. The functional area of the polypeptide exposed to the MBD can combine with 5-methyl cytosine fragments and can combine directly with the 5-methyl cytosine surface [13]. Therefore, in combination with column chromatography, the 5-methyl cytosine is directly separated from other strands.

## 4. Bisulfite-Free and Enzyme-Free Techniques

**Figure 4:**
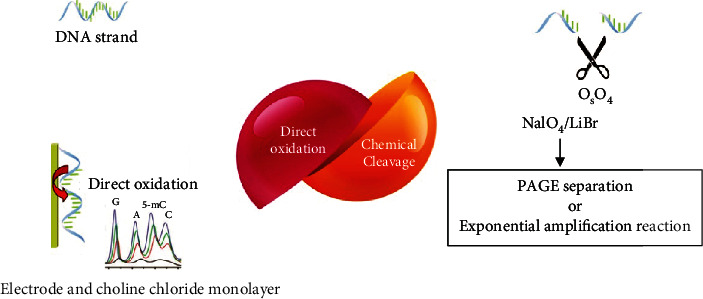
Two methods of analysis without bisulfite and enzyme. One measures the signals produced in the oxidation reaction, and the other measures the broken target pieces by performing chemical reactions.

Although the bisulfite conversion methods and biological approaches have shown specificity to characterize the position of DNA methylation, there are still issues of reaction efficiency, costs of analysis, and operational complexity in the analysis of 5-methyl cytosine. An enzyme- and bisulfite-free method for DNA methylation should overcome these constraints and have many advantages such as speed, convenience, and low cost (Figure 4).

### 4.1. Analysis Based on Direct Oxidation

Thymine and 5-methyl cytosine are oxidized by osmium tetroxide in dual C5-C6 bonds. Therefore, there is a potential proper way to use direct electrochemical oxidation of pyrimidine for the analysis of DNA methylation [65]. In 2010, an electrochemical method was proposed for the general analysis of DNA methylation [66]. The choline chloride monolayer-supported multiwalled carbon nanotubes (MWCNTs) (MWCNTs/Ch/GCE) were made [67]. The MWCNTs provide significant electrocatalytic activities for DNA bases. All of the bases of purine, guanine, pyrimidine, thymine, adenine, cytosine, and 5-methyl cytosine are detected by their unique oxidation signals [68]. In the following years, an advanced method was designed to improve the capacity of determining the mixing bases using overoxidized polypyrrole guided by MWCNTs/GCE (PPyox/MWCNTs/GCE) [69]. This technique has an extraordinary specificity and precision and can be used to rapidly determine the mixing bases without using the enzyme, probe, or bisulfite.

### 4.2. Analysis Based on the Chemical Decomposition of Oxidation

Recently, several new chemical methods based on chemical oxidation decomposition have been proposed to separate 5-methyl cytosine [70]. Inspired by the capability to separate 5-methyl cytosine from cytosine by OsO_4_, Yamada et al. [71] developed a method for detecting 5-methyl cytosine through light-sensitive oxidation using the 2-methyl-1,4-naphthoquinone-chromophore. Since then, an advanced method has been reported by using NaIO_4_/LiBr [72]. The C5-C6 dual bond of 5-methyl cytosine in the DNA is selectively oxidized by NaIO_4_/LiBr and then broken down using the hot piperidine method [73]. The DNAs chemically decomposed are analyzed by electrophoresis of the polyacrylamide gel (PAGE), and the status of DNA methylation is easily achieved [74]. The chemical cut-off method shows high efficiency for detecting the methylation sites. However, its sequence nonselectivity and undesirable sensitivity prevent its application in the genetic methylation analysis.

## 5. Conclusion

Many techniques have been developed to determine the status of DNA methylation over the past two decades. These diverse methods have three stages, providing the possibility of selecting and analyzing the methylated DNA from nonmethylated DNA in the target genome. In the first stage, the methylated and nonmethylated fragments can be distinguished through three main groups from the pretreatment methods, including the conversion of bisulfite, digestion with methylation-sensitive restriction enzymes, and dependence of the methylated DNA. The bisulfite treatment, extensively used in various platforms, relies on the conversion of nonmethylated cytosine to uracil and the nonalteration of the methylated cytosine. In this method, the methylated and nonmethylated fragments are identified by differences in the sequence. The restriction enzymes, sensitive and insensitive to methylation, including Hpa II and Msp I, are used to measure the methylation of genes. The Hpa II is stopped in the presence of 5-methyl cytosine in the four-nucleotide CCGG sequence, while the Msp I is not affected by DNA methylation. Therefore, the analysis of cut-off DNA products can be used for the detection of CpG methylation in specific genomic locations. The physical separation can be done by using antibodies and proteins binding to methylated cytosine.

The second stage is the replication of treated DNA with PCR. The last step is DNA methylation using various techniques like MS-PCR, COBRA, bisulfite-free sequencing, or microarray analysis. These techniques have been developed to determine the status of DNA methylation in general, specific genes, or genomic-wide surfaces. Initially, the analyses focused on the approaches of special locations. But now, the analyses can be performed on a large genomic scale using the new powerful technologies, including the DNA methylation microarrays and broad genomic free-bisulfite sequencing. However, recent advances have been made in the DNA methylation analysis but some barriers limit the application of these methods. (i) Significant errors during PCR amplification, DNA extraction, and the process of bisulfite conversion led to false results. (ii) Both PCR and bisulfite tests are time-consuming and boring. (iii) High-throughput technologies are costly and very complicated to run in standard laboratories. The appropriate approach in DNA methylation analysis depends on the study objectives. No DNA methylation analysis method will be appropriate for all applications. By knowing the type of information presented in each method, the researchers will be able to choose the most appropriate method based on their research needs.

## Data Availability

The datasets used in this study are available from the corresponding author on reasonable request.

## Abbreviations

MS-PCR: Methylation-specific PCR
Ms-SnuPE: Methylation-sensitive single-nucleotide primer extension
RRBS: Reduced representation bisulfite-sequencing
MS-DGGE: Methylation-specific denaturing gradient gel electrophoresis
MS-MCA: Methylation-specific melting-curve analysis
COBRA: Combined bisulfite-restriction analysis
MREs: Methylation-sensitive restriction enzymes
RLGS: Restriction-landmark genomic scanning.

## Additional Points

*Transparency Document*. The transparency document associated with this article can be found in the online version.
